# Editorial Perspective: Overdiagnosis of ADHD? Here we go again

**DOI:** 10.1111/camh.70075

**Published:** 2026-02-22

**Authors:** Anita Thapar

**Affiliations:** ^1^ Cardiff University Cardiff UK; ^2^ University of Bristol Bristol UK

**Keywords:** ADHD, mental health, overdiagnosis, NHS‐E ADHD Taskforce report, early intervention

## Abstract

There is growing preoccupation with whether ADHD is overdiagnosed. That question has already been addressed for the United Kingdom in the recently published National Health Service (NHS)‐England independent ADHD Taskforce Report. The administrative prevalence of ADHD in the United Kingdom is lower than the population rate. Regardless of prevalence or country, I argue why a focus purely on diagnosis is unhelpful and overly narrow. A diagnosis is helpful for clinical decision making but, on its own, has multiple limitations. I argue the case for why a needs‐based, stepped care, holistic approach is preferable and provide evidence of what sorts of timely support are likely to be effective. The time has come for constructive rather than divisive dialogue on how best to help children and young people.

There is growing preoccupation about whether there is overdiagnosis of ADHD as well as mental health disorders, especially among youth. For example, recently in the United Kingdom, the Government has announced a review into the evidence on overdiagnosis of ADHD and mental health problems. In my view, this focus is unhelpful for many reasons.

## A focus on addressing needs and functioning is preferable

While diagnoses are helpful for clinical decision‐making, such as selecting effective treatments, estimating prognosis and for communication, diagnosis alone has limitations. The under‐ versus overdiagnosis debate does not generate solutions for improving the mental health and well‐being of children and young people and takes an unduly narrow, specialist, medically focused approach when a broader, stepped approach based on needs and severity is preferable.

## ADHD and many mental health conditions lie on a continuum

A focus on under versus overdiagnosis fails to recognise the underlying nature of ADHD and many mental health conditions that do not exist as yes/no categories. The provision of support should mirror this. For ADHD, risks of adverse outcomes and impaired functioning increase across the continuum of symptom severity and there is no clear‐cut threshold at which adverse outcomes appear (Thapar & Cooper, 2016). In this respect, ADHD is like blood pressure and hypertension or blood lipids and hyperlipidaemia. While medication has been shown to be an effective ADHD treatment, at the boundaries of a diagnostic threshold, the risks of medication may outweigh benefits. However, there are multiple other nonpharmacological interventions available for those with milder ADHD as outlined in NICE guidance (National Institute for Health and Care Excellence, 2013).

## Hazards of trivialising ADHD

This is a concern given the long history of underrecognition, underidentification and undertreatment as well as scepticism that ADHD is a valid diagnostic entity. This is despite evidence that ADHD can lead to mental and physical ill‐health, a shortened lifespan not dissimilar to diabetes, educational failure, unemployment, social difficulties, self‐harm and suicide, substance misuse and early entry into the criminal justice system (ADHD Taskforce, 2025). Yet, we have excellent evidence that such risks can be reduced, and available treatments are more effective than many interventions in general medicine such as lipid lowering drugs and antihypertensive medication (Leucht, Hierl, Kissling, Dold, & Davis, 2012). It is intriguing to me that while antiobesity treatments have been welcomed as they cut risks for those with obesity, there is a different standard for those with ADHD despite the well‐documented risks and the availability of so many effective treatments with good effect size.

## This debate is highly discriminatory and divisive

Is there anywhere else in the health service where research evidence is repeatedly disregarded? Those with ADHD, like those with mental health problems, already face enormous inequalities, discrimination, disbelief and injustice. Why further compound these inequalities? Social and health support for those with ADHD lags far behind those with a physical health condition as outlined in the recent ADHD Taskforce report (ADHD Taskforce, 2025). National Health Service (NHS) waiting times in the United Kingdom are not equitable for those with ADHD despite the availability of effective interventions across the spectrum of severity.

## Why not focus on constructive ways forward?

In the last decade, rates of some but not all types of mental health problems have increased. High quality research in England observed sharp rises in the population prevalence of youth emotional problems and eating disorders but not in ADHD (National Health Service, 2020). Those findings have been widely replicated. However, rates of referral to specialist services for ADHD have increased enormously. These secular increases are not restricted to the United Kingdom but have been observed across Europe and the USA, although youth in the United Kingdom appear to fare especially poorly and this situation has worsened after the Covid‐19 pandemic. There are many hypotheses as to what might explain these rises in mental ill‐health, but some drivers such as austerity, economic inequality, educational environments and opportunities, as well as youth unemployment among others can be addressed.

## NHS England independent ADHD Taskforce recommendations

We highlighted multiple different solutions in the ADHD taskforce report that could easily be extended to mental health and are not enormously expensive. The taskforce was supported by clinicians, leading UK scientists and those with lived experience.

### Over‐or underdiagnosis of ADHD

The taskforce began by examining the rates of ADHD diagnosis in the United Kingdom. Robust evidence from epidemiological studies, prescription and NHS data all converged to show that in the United Kingdom, ADHD when defined rigorously, is underrecognised, underdiagnosed and undertreated (ADHD Taskforce, 2025). That is not the case for all countries, but UK treatment rates are well below most other higher income countries. Unlike emotional problems and eating disorders, the prevalence of ADHD in the community has not changed across the last few decades. The reasons for the surge in ADHD referrals across Europe are likely multiple. They include changes in diagnostic criteria, reduced stigma, increased awareness, the impact of the Covid‐19 pandemic on the mental health of neurodivergent people, among other reasons.

Research evidence also has evolved as have available treatments. We do not question practice or service need changes for physical health (e.g. hypercholesteraemia, ischemic heart disease, cancers and obesity) driven by new evidence. When I trained in psychiatry in the late 1980s, the core ADHD concept was known as hyperkinetic disorder and described a rare condition that primarily affected boys, was restricted to childhood and was always easily observable to others. Subsequent research has demonstrated that ADHD also affects females, extends to adult life and across the spectrum of cognitive ability and its features can be masked in some.

This has meant that many with ADHD, especially females, were missed or misdiagnosed as having depression or anxiety (Martin et al., 2025). For others, impairment in functioning due to ADHD symptoms only becomes evident in early adulthood as demands increase and parental support and scaffolding reduces. That then leads to later help‐seeking for ADHD. Those who were not identified in childhood now are coming forward in adult life and adult services have had to rapidly evolve to manage this. Again, this catch‐up is being observed across other European countries and service capacity adjusted accordingly including a much greater involvement of GPs for adult ADHD in some countries (e.g. Australia).

In the United Kingdom, this has led to unacceptably long wait times with well‐documented inequality to service access. ADHD remains unrecognised or untreated disproportionately in disadvantaged and minority groups.

### Transformation of ADHD support needs to cross systems

ADHD is not the remit of health care alone and integration of support for ADHD is needed across health, education, social work, criminal justice and the workplace with training to recognise and support ADHD that crosses sectors and professional groups.

### Needs‐based, upstream prevention and early intervention

#### Preschool

For many people with ADHD, support is too little and too late. Intervention needs to start early in the preschool years before adverse outcomes arise. This includes family support and evidence‐based parenting interventions including ones that are ADHD or neurodivergent‐focused. Early years support has increasingly been cut across the United Kingdom especially since 2010. Follow‐up studies, however, have shown that children living in Sure Start areas displayed better health and educational achievement with fewer special educational needs by adolescence, with clear‐cut cost benefits to the government. This highlights the importance of such early life interventions, especially for the most disadvantaged families (Hayre, Pearce, Khera, Lunn, & Ford, 2025).

#### School

We also recommended universal and targeted support for mental health, ADHD and neurodivergence in schools. Again, such support should not be based on presumed diagnoses or wait for a clinical assessment but rather address needs and functioning. School and education policies impact on children's mental health and ADHD outcomes, so education is a crucial sector for supporting children and young people. However, explicit joint working with neurodevelopmental and child and adolescent mental health services is also important to enable consultation and rapid access to clinical assessment and treatment for those who need this.

#### Integrated youth services

Young people aged 12–25 years are of special concern as rates of mental health problems have increased most sharply in this age group. It also spans multiple social, educational and service transitions. Outside the United Kingdom, many countries have created integrated youth services that span this age group. For example, the model evaluated in Canada (Henderson, Szatmari, Cleverley, et al., 2025) is needs—rather than diagnosis—focused and is codesigned with young people. These services provide a one‐stop shop of holistic care for young people and bring together primary and secondary mental and physical health including sexual health, education, employment and vocational services, social services, peer support and the voluntary sector. This type of service crosses diagnostic silos, and, compared to treatment as usual, young people are seen more quickly and use fewer psychiatry resources. Many of the separate components of integrated youth services already exist in the United Kingdom but have not been integrated nor services codesigned with young people.

### Redesign and transformation of ADHD services

This also cannot be ignored because of escalating costs and historic neglect of ADHD with an urgent need to address growing wait times. One key barrier to transformation is the growth of diagnostic silos between ADHD, neurodivergence and mental health that need to be disrupted across clinical practice, services and budgets given overlaps are so typical. Another is that we are failing to recognise that ADHD is a common condition affecting 3–5% of the population. Thus, there is the need to expand an ADHD‐capable workforce beyond a secondary care, superspecialist model and improve efficiencies through digitisation. Rather than services that focus only on treating diagnosis, we recommended a stratified stepwise approach to care as there is for so many conditions in the NHS. This could begin with psychoeducation, lifestyle changes and environmental adjustments that do not require a definitive diagnosis or delivery from specialist CAMHS/ND services. However, training across sectors and professional groups is crucial to enable this. Another concern is ensuring uniform quality and accountability of ADHD services across the nation and the collection of high‐quality data. For those with lived experience of ADHD, the authenticity of their diagnoses is often questioned, and this further disrupts continuity of care; quality assurance across all providers is essential.

## Conclusion

My personal view is that the current focus on whether there is overdiagnosis of ADHD and mental health is unhelpful and not constructive. While this may not be the intention, many view the focus as an excuse to avoid action. We already know that ADHD is underrecognised, underdiagnosed and undertreated in the United Kingdom and have good data on which types of mental health conditions have risen in prevalence. We also know that adolescents and young people are the most affected. Children and adults continue to experience unacceptably long wait times for ADHD support, thereby increasing the risks of costly, secondary adverse outcomes. The ADHD taskforce, at considerable effort and time, provided several recommendations that could be implemented to improve efficiencies and outcomes including a shift to a needs‐led stepped care model of assessment and intervention. The time has come to now move past the repeated debate about diagnosis and focus on timely and appropriate support of children and young people. The research evidence as to what works is already there. It simply needs political will to implement change.

## Conflicts of interest statement

A.T. was Chair of the NHS‐E independent ADHD taskforce and has also served as cochair of the Welsh Government Ministerial Advisory Group for neurodivergence and Department for Education (England) neurodivergence task and finish group. She is currently a member of the Welsh Government Adult ADHD Task and Finish Group.

## Funding information

No funding to declare in relation to this article.

## Ethics statement

No ethical approval was required for this article.

## Data Availability

Data sharing is not applicable to this article as no datasets were generated or analysed.

## References

[camh70075-bib-0001] ADHD Taskforce (2025). Report of the independent ADHD Taskforce. London: NHS England.

[camh70075-bib-0002] Hayre, J. , Pearce, H. , Khera, R. , Lunn, A.D. , & Ford, J.A. (2025). Health impacts of the sure start programme on disadvantaged children in the UK: A systematic review. BMJ Open, 15, e089983. 10.1136/bmjopen-2024-089983 PMC11842985 39979044

[camh70075-bib-0003] Henderson, J. , Szatmari, P. , Cleverley, K. , Ma, C. , Hawke, L.D. , Cheung, A. , … & Shan, D. (2025). Integrated collaborative care for youths with mental health and substance use challenges: A randomized clinical trial. JAMA Network Open, 8, e259565. 40358950 10.1001/jamanetworkopen.2025.9565 PMC12076176

[camh70075-bib-0004] Leucht, S. , Hierl, S. , Kissling, W. , Dold, M. , & Davis, J.M. (2012). Putting the efficacy of psychiatric and general medicine medication into perspective: Review of meta‐analyses. The British Journal of Psychiatry, 200, 97–106. 22297588 10.1192/bjp.bp.111.096594

[camh70075-bib-0005] Martin, J. , Rouquette, O.Y. , Langley, K. , Cooper, M. , Sayal, K. , Ford, T. , … & Thapar, A. (2025). Antecedents and outcomes of a late attention deficit hyperactivity disorder (ADHD) diagnosis in females. *MedRxiv*. 10.1101/2025.07.07.25330613 PMC7618889 41804225

[camh70075-bib-0006] National Health Service . (2020). Mental Health of Children and Young People in England, 2020: Wave 1 follow up to the 2017 survey ‐ NHS Digital. Mental Health of Children and Young People in England, 2020: Wave 1 follow up to the 2017 survey. Available from: https://digital.nhs.uk/data‐and‐information/publications/statistical/mental‐health‐of‐children‐and‐young‐people‐in‐england/2021‐follow‐up‐to‐the‐2017‐survey [last accessed December 3, 2021].

[camh70075-bib-0007] National Institute for Health and Care Excellence . (2013). Attention deficit hyperactivity disorder: Diagnosis and management of ADHD in children, young people and adults. Available from: http://www.nice.org.uk/guidance/cg72. [last accessed December, 2025].

[camh70075-bib-0008] Thapar, A. , & Cooper, M. (2016). Attention deficit hyperactivity disorder. The Lancet, 387, 1240–1250. 10.1016/S0140-6736(15)00238-X 26386541

